# Thioredoxin-1 Protects Bone Marrow-Derived Mesenchymal Stromal Cells from Hyperoxia-Induced Injury In Vitro

**DOI:** 10.1155/2018/1023025

**Published:** 2018-01-21

**Authors:** Lei Zhang, Jin Wang, Yan Chen, Lingkong Zeng, Qiong Li, Yalan Liu, Lin Wang

**Affiliations:** ^1^Hepatic Surgery Center, Tongji Hospital, Tongji Medical College, Huazhong University of Science and Technology, Wuhan, Hubei 430030, China; ^2^Department of Pediatrics, Union Hospital, Tongji Medical College, Huazhong University of Science and Technology, Wuhan, Hubei 430022, China; ^3^Department of Neonatal, Wuhan Children's Hospital (Wuhan Maternal and Child Healthcare Hospital), Tongji Medical College, Huazhong University of Science and Technology, Wuhan, Hubei 430014, China

## Abstract

**Background:**

The poor survival rate of mesenchymal stromal cells (MSC) transplanted into recipient lungs greatly limits their therapeutic efficacy for diseases like bronchopulmonary dysplasia (BPD). The aim of this study is to evaluate the effect of thioredoxin-1 (Trx-1) overexpression on improving the potential for bone marrow-derived mesenchymal stromal cells (BMSCs) to confer resistance against hyperoxia-induced cell injury.

**Methods:**

80% O_2_ was used to imitate the microenvironment surrounding-transplanted cells in the hyperoxia-induced lung injury *in vitro*. BMSC proliferation and apoptotic rates and the levels of reactive oxygen species (ROS) were measured. The effects of Trx-1 overexpression on the level of antioxidants and growth factors were investigated. We also investigated the activation of apoptosis-regulating kinase-1 (ASK1) and p38 mitogen-activated protein kinases (MAPK).

**Result:**

Trx-1 overexpression significantly reduced hyperoxia-induced BMSC apoptosis and increased cell proliferation. We demonstrated that Trx-1 overexpression upregulated the levels of superoxide dismutase and glutathione peroxidase as well as downregulated the production of ROS. Furthermore, we illustrated that Trx-1 protected BMSCs against hyperoxic injury via decreasing the ASK1/P38 MAPK activation rate.

**Conclusion:**

These results demonstrate that Trx-1 overexpression improved the ability of BMSCs to counteract hyperoxia-induced injury, thus increasing their potential to treat hyperoxia-induced lung diseases such as BPD.

## 1. Introduction

Bronchopulmonary dysplasia (BPD) is a chronic lung disease that typically occurs in very low-birth-weight premature infants following supplemental oxygen therapy and mechanical ventilation. An increase in the survival rate of these extremely premature infants has been associated with an increased incidence of BPD. BPD is a multifactorial disease, and hyperoxia, or oxygen toxicity, is known to play a key role in its pathogenesis [1]. Oxygen toxicity is believed to be mediated by the production and accumulation of reactive oxygen species (ROS), such as superoxide (O_2_^−^), hydrogen peroxide (H_2_O_2_), and hydroxyl radicals (•OH), to levels exceeding the capacity of the antioxidant defense mechanisms [2]. It is well known that ROS is required in a myriad of physiological reactions, cell fate decisions, and signal transduction pathways. However, overwhelming accumulation of ROS will trigger severe oxidative stress through enzyme oxidation, protease inhibition, DNA synthesis inhibition, and lipid peroxidation, which commits cells to necrosis or apoptosis [3].

Currently, no effective treatments beyond supportive therapies are available for BPD. Stem cell-based treatment via tissue engineering is currently an increasing focus of research [4]. As bone marrow-derived mesenchymal stromal cells (BMSCs) come from an autologous source and are easy to isolate and amplify [4], they are the ideal seed cells for tissue engineering across broad tissue types such as the liver [5], bone [6], lung [7], heart [8], and kidney [9]. Recently, studies have shown that lung repair by BMSC therapy could be a promising and novel therapeutic modality for attenuating BPD severity [10, 11]. These studies have demonstrated that BMSCs enhance lung repair by direct regeneration or through secreting paracrine factors. However, several studies confirmed the low survival and poor engraftment rates of MSCs in recipient lungs, which greatly limits their therapeutic efficacy, as survival of the transplanted cells in the pathological environment is critical for their beneficial effects [12, 13]. Hence, one major focus in the field is to explore the mechanism involving the BMSC injury in pathological environment and to develop strategies to enhance BMSC survival and engraftment rates.

Thioredoxin (Trx), a ubiquitous small protein (12 kDa) containing a redox-active dithiol/disulfide at a highly conserved active site, was originally identified as a hydrogen donor for ribonucleotide reductase in *Escherichia coli* [14]. There are two main thioredoxins: thioredoxin-1 (Trx-1), a cytosolic form, and thioredoxin-2 (Trx-2), a mitochondrial form. Trx, along with Trx reductase (TrxR) and nicotinamide adenine dinucleotide phosphate (NADPH), has been shown to catalyze protein disulfide reduction and is thought to be a strong ROS scavenger [15]. Trx-1 participates in redox reactions through reversible oxidation of its dithiol active center to disulfide which catalyzes dithiol-disulfide exchange reactions involved in many thiol-dependent processes [16]. By this way, Trx-1 acts on oxidized, therefore inactive, proteins by reducing them and restoring their functionality. Recent studies have shown that Trx-1 not only regulates the cellular redox balance by scavenging intracellular ROS ingredients, such as hydrogen peroxide (H_2_O_2_), but also has other biological activities, including regulation of cell growth, transcription factors, gene expression, apoptosis, and immune regulatory effects [17–19]. Our previous studies suggest that Trx protects alveolar epithelial cells from hyperoxia-induced injury by reducing ROS generation, elevating antioxidant activities, and regulating the MAPK and PI3K-Akt pathways [20].

Based on previous studies from others and our own work, we hypothesize that BMSCs suffer severe injury under hyperoxic conditions and that increased Trx-1 expression in BMSCs may serve to counteract the negative effects of hyperoxia-induced cell injury. To better understand the mechanism of Trx-1, we also looked into the signaling pathways mediated by it in hypoxia-induced cell injury. Our data may provide a new perspective in the development of BMSC therapeutic strategies.

## 2. Materials and Methods

### 2.1. BMSC Culture

All studies were performed under the approval of the Ethics Committee of the Animal Facility of Huazhong University of Science and Technology. BMSCs were isolated from the bone marrow of 6- to 7-week-old male Sprague-Dawley rats (provided by Tongji Medical College, Huazhong University of Science and Technology, Wuhan, China) according to the previously described method with some modifications [11, 21, 22]. Briefly, bone marrow cells were flushed from rat tibias and femurs, suspended by pipetting, and filtered via nylon mesh (70 *μ*m). The collected mononuclear cells were washed three times with Dulbecco's phosphate-buffered saline (DPBS). The cells were suspended in a culture medium (DMEM medium containing 10% FBS, 0.02% sodium bicarbonate, 2 mM L-glutamine, 15 mM HEPES buffer, 100 units/mL penicillin, and 100 *μ*g/mL streptomycin) and incubated at 37°C in an atmosphere of 95% humidified air and 5% CO_2_ for 24 h. The medium was exchanged with a fresh culture medium in an attempt to deplete the nonadherent cells. When adherent cells were grown to approximately 75% confluency, they were trypsinized and reseeded at a density of 10^5^ cells/cm^2^.

### 2.2. Phenotypic Analysis of BMSCs

Flow cytometric analysis was performed to characterize the phenotype of BMSCs. Cells were suspended in 100 *μ*L DPBS supplemented with 2% FBS. Phycoerythrin- (PE-) coupled antibodies against CD29 (eBiosciences, cat. no. 12-0291, San Diego, CA, USA), CD34 (Santa Cruz Biotechnology, cat. no. sc-74499), CD44 (Santa Cruz Biotechnology, cat. no. sc-7297), CD45 (Santa Cruz Biotechnology, cat. no. sc-1178), and CD90 (Santa Cruz Biotechnology, cat. no. sc-53456) were added separately, followed by incubation at 4°C for 30 minutes. For the detection of cell surface antigens CD105 and CD73, cells were incubated with the first antibodies against CD105 (Abcam, cat. no. ab156756) and CD73 (Abcam, cat. no.ab175396) for 1 hour at 4°C, washed, and then incubated for 1 hour at 4°C with Alexa Fluor 647-conjugated second antibodies (Invitrogen, cat. nos. A-21235 and A-21244). Irrelevant isotype-identical antibodies served as negative control. After washing, more than 10,000 cells were acquired using a FACS Calibur (Becton Dickins) flow cytometer and analyzed with FlowJo software (FlowJo LLC, Ashland, Oregon, USA).

### 2.3. BMSC Differentiation

To confirm that our cultured cells have multipotent potential, we tested BMSC P3 cultures for their ability to undergo differentiation into osteocytes and adipocytes as previously described [23, 24]. Briefly, osteogenic differentiation was induced by incubating BMSCs with an osteogenic medium (RASMX-90021; Cyagen Biosciences, Guangzhou, China), and adipocyte differentiation was induced by maintaining BMSCs in an adipocyte differentiation medium (RASMX-90031; Cyagen Biosciences). After 21 days of differentiation, cells were fixed and stained with alizarin red and oil red O separately.

### 2.4. Transfection

In order to achieve high efficiency of introduction and subsequent stable expression of rat Trx-1 in BMSCs, a lentiviral vector was employed. Briefly, the third passage of BMSCs was transfected with lentiviral vectors carrying Trx-1 and green fluorescent protein (GFP) (pCDH-CMV-Trx-1-EF1*α*-copGFP) or a lentiviral vector carrying only GFP (pCDH-CMV-MCS-EF1*α*-copGFP) using the Lipofectamine 2000 transfection reagent according to the manufacturer's instructions (Invitrogen, Carlsbad, CA, USA). The transfected BMSCs were termed BMSCs-p (lentiviral vector only carried GFP) and BMSCs-Trx-1 (lentiviral vectors carried Trx-1 and GFP). The recombinant plasmids were constructed and identified by Wuhan Transduction Bio Co. Ltd. (Wuhan, China). Stably transfected cells were then selected by incubation in the fresh FBS-supplemented DMEM culture medium containing 500 *μ*g/mL G418. BMSCs not subjected to transfection served as control cells. The expression of Trx-1 was detected by reverse transcriptase polymerase chain reaction (RT-PCR) analysis and Western blot analysis.

### 2.5. Hyperoxia and Normoxia Treatment

Cells were seeded into 6-, 24-, or 96-well cell culture plates overnight. The next day, cells were placed in hyperoxia (80% O_2_, 5% CO_2_) or normoxic (21% O_2_, 5% CO_2_) environment as previously described [20, 25, 26]. The concentration of O_2_ was monitored in real time with a digital oxygen monitor (Hengaode, Beijing, China). Cells were harvested at 0, 12, 24, and 48 hours.

### 2.6. Cell Proliferation Assay

In order to determine the influence of Trx-1 overexpression on BMSC proliferation, cell proliferation assays were performed using a Cell Counting Kit-8 (CCK-8, Dojindo, Japan) according to manufacturer's protocol. Cells were seeded into a 96-well plate in triplicate at 5000 cells/well and cultured overnight. Cells were then exposed to hyperoxic or normoxic conditions described above. After the exposures, the number of cells per well was measured by the 450 nm absorbance of reduced WST-8 (2-(2-methoxy-4-nitrophenyl)-3-(4-nitrophenyl)-5-(2, 4-sulfophenyl)-2H-tetrazolium, monosodium salt) at the indicated time points [27, 28]. In addition, a blank control well was set containing only the culture medium.

### 2.7. Cell Apoptosis Assay

Apoptosis was measured by flow cytometry after annexin V-PE/7-AAD staining (BD Pharmingen, USA) according to manufacturer's instructions. Briefly, the treated cells were harvested with Accutase solution (Gibco/Life Technologies, cat. no. A11105-01), washed twice with cold PBS, and suspended in 1x binding Buffer. Then, the cells were labeled with annexin V-PE and 7-AAD for 15 minutes at room temperature in the dark. Apoptosis-positive control cells were placed in 50°C water bath for 5 minutes. Finally, the cells were subjected to flow cytometry analysis using a FACS Caliber flow cytometer (BD Biosciences, CA) within 30 minutes.

### 2.8. Measurement of Intracellular ROS Accumulation

ROS production was measured with CellROX® deep red reagent from Molecular Probes (Eugene, OR, USA). The CellROX deep red reagent is a fluorogenic probe designed to reliably measure ROS in living cells. The cell-permeable CellROX deep red dye is nonfluorescent while in a reduced state and becomes fluorescent upon oxidation by reactive oxygen species with absorption/emission maxima at ~644/665 nm [29]. After treatment, cells were incubated at 37°C for 30 minutes in complete DMEM with 5 mM CellROX deep red reagent. Then, the medium was removed and the cells were washed 3 times with PBS. Cells were collected and suspended in PBS. Fluorescence was immediately measured using FACS analysis, and values were reported as mean fluorescence intensity.

### 2.9. Hydrogen Peroxide Assay

The level of intracellular H_2_O_2_ was measured using Hydrogen peroxide assay kit (Beyotime Institute of Biotechnology, China) as described previously [30, 31]. In this assay system, ferrous ions (Fe^2+^) are oxidized to ferric ions (Fe^3+^) by H_2_O_2_. Then, the Fe^3+^ and the indicator dye xylenol orange form a purple complex, which is measurable with a microplate reader at a wavelength of 560 nm.

According to the manufacturer's protocol, cells were lysed using the lysis buffer solution supplied in the kit at a ratio of 100 *μ*L per 10^6^ cells. After centrifugation at 12,000*g* for 5 minutes, the supernatants were collected. 50 *μ*L of each supernatant sample was put into 100 *μ*L of test solution, and the mixture was incubated for 20 minutes at room temperature. Finally, the absorbance at 560 nm was measured using a microplate reader (Elx 800; BioTek). The level of H_2_O_2_ in cells was determined using a standard curve prepared by plotting the average blank-corrected 560 nm measurement for each standard.

### 2.10. Caspase 3 Activity Assay

Caspase 3 activity was measured using Caspase 3 Activity Assay kit (Beyotime Biotechnology, Nanjing, China) following the manufacturer's instructions [32]. After being subjected to treatment described above, cells were detached from plates, washed with PBS, and centrifuged at 1200 rpm for 5 minutes at 4°C for cell collection and lysis. Caspase 3 activity was detected using the specific fluorogenic substrates Ac-DEVD-pNA; the absorbance at 405 nm was measured using a microplate reader (Elx 800; BioTek).

### 2.11. RNA Isolation and Real-Time PCR

RNA samples were prepared using the RNAiso plus kit (Takara Bio Inc., Kusatsu, Shiga, Japan) according to manufacturer's instructions. Total RNA (1 *μ*g) was used to reverse transcribed cDNA using iScriptTM cDNA synthesis kit (Takara Bio Inc.) according to manufacturer's instructions. Real-time PCR was performed using iQ SYBR Green Supermix (Bio-Rad Inc. Laboratories, Hercules, CA, USA). Amplification, detection, and data analysis were performed with the iCycler real-time detection system (Bio-Rad Inc.). GAPDH was used as the endogenous control. Specific primer sets for Trx-1 and GAPDH were obtained from Invitrogen. The relative expression level of Trx-1 was determined using the 2^−delta delta Ct^ analysis method. The primer sequences used for PCR were as follows: Trx-1, forward 5′-TTCTTTCATTCCCTCTGTG-3′ and reverse 5′-TCCGTAATAGTGGCTTCG-3′; GAPDH, forward 5′-GTTCTTCAATACGTCAGACATTCG-3′ and reverse 5′-CATTATCTTTGCTGTCACAAGAGC-3′.

### 2.12. Western Blot Analysis

Cell protein levels of Trx-1, apoptosis-regulating kinase-1 (ASK1), phosphorylated ASK1 (p-ASK1), p38, and phosphorylated p38 (p-p38) were analyzed by Western blotting, using *β*-actin as an internal reference. Briefly, total proteins were extracted using a protein extraction kit (KGP2100; KeyGEN Biotech, Nanjing, China), quantified by BCA protein assay (Guge Bio, Wuhan, China), electrophoresed on SDS-PAGE gels, and electrotransferred to PVDF membrane by wet transfer (Bio-Rad). Membranes were blocked for 1 hour with 5% skim milk and incubated overnight at 4°C with the primary antibodies. The anti-ASK1 antibody came from Abcam (cat. no. ab131506), anti-p-ASK1 antibody came from Sigma (cat. no. SAB4504337), and all other antibodies came from Cell Signaling Technology Inc., Danvers, MA, USA. Membranes were washed in TBS/0.1% Tween-20 to remove excess primary antibodies. The membranes were then incubated for 1 hour with the secondary antibodies (Cell Signaling Technology Inc.). After three washes in TBS/0.1% Tween-20, the protein bands were visualized using an enhanced chemiluminescence kit according to the manufacturer's instructions (ECL; Pierce Biotechnology Inc., Rockford, IL, USA). Densitometry was measured using “ImageJ” analysis software.

### 2.13. Antioxidant Enzyme Activity Measurements

The activities of total superoxide dismutase (T-SOD), catalase (CAT), and glutathione peroxidase (GSH-Px) were estimated by the test kits according to the manufacturers' instructions. T-SOD assay kit was purchased from Nanjin Jiancheng Biotechnology Co. Ltd. (Nanjing, Jiangsu, China) [33]. The GSH-Px assay kit and CAT activity assay kit were purchased from Beyotime Institute of Biotechnology (Shanghai, China) [34, 35]. Briefly, the cells were washed with PBS and lysed using cell lysis buffer. Cell lysates were then centrifuged at 10,000*g* for 5 minutes at 4°C, and the supernatants were collected to determine enzyme activities. These assays were performed on the Elx800 microplate reader at 550 nm for T-SOD, 520 nm for CAT, and 340 nm for GSH-Px, respectively. The values were normalized and expressed as units per mg protein, based on protein concentrations determined using BCA protein assay (Guge Bio).

### 2.14. Enzyme-Linked Immunosorbent Assay (ELISA)

After treatment, culture supernatants were collected and spun at 300*g* for 10 minutes to remove cellular debris. The levels of keratinocyte growth factor (KGF), hepatocyte growth factor (HGF), and epidermal growth factor (EGF) were determined by employing ELISA kits (R&D System, Minneapolis, MN, USA) according to the manufacturer's protocol. Each sample was analyzed in triplicate.

### 2.15. Statistical Methods

All data were reported as mean ± standard deviations (mean ± SD) and analyzed by using SPSS 18.0 (SPSS Inc., Chicago, IL, USA). Data were analyzed statistically using ANOVA or Student's *t*-test. Significance was accepted at *P* < 0.05.

## 3. Results

### 3.1. Characterization of BMSCs

**Figure 1:**
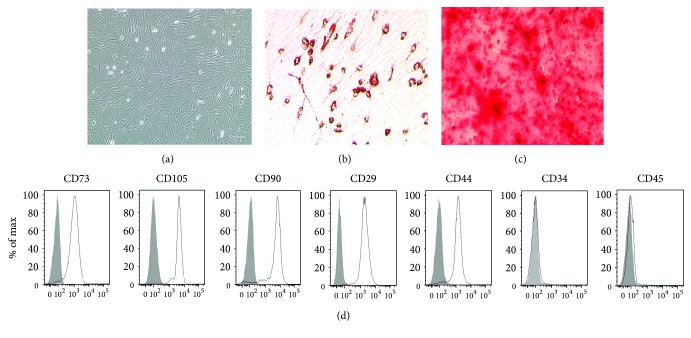
Characterization of rat bone marrow-derived mesenchymal stromal cells (BMSCs). (a) The plastic-adherent cells demonstrated a homogeneous fibroblast-like and spindle-shaped morphology. Original magnification, ×100. (b) Adipogenic differentiation of BMSCs stained with oil red O. Original magnification, ×200. (c) Osteogenic differentiation of BM-MSCs stained with alizarin red. Original magnification, ×400. (d) FACS analysis demonstrated expression of markers attributed to BMSCs. The cells were devoid of hematopoietic cells as indicated by the lack of CD45 and CD34. The MSC-specific markers, CD29, CD44, CD73, CD105, and CD90 were strongly expressed on the cells.

The BMSC cultures were observed by using an inverted light microscope. BMSCs are plastic-adherent cells that showed a flattened and spindle-shaped morphology. About 10 days later, the primary cultured cells developed to clusters and could be used for subculture. After two to three passages, BMSCs demonstrated a homogeneous fibroblast-like, spindle-shaped morphology. The morphological features of the BMSCs are shown in Figure 1(a). To verify the pluripotent capacity of the cultured cells, we cultured the cells in adipogenic or osteogenic differentiation induction media for 21 days. Differentiation toward these cell lineages was demonstrated by oil red O and alizarin red staining, respectively (Figures 1(b) and 1(c)). As illustrated in Figure 1(d), the BMSC population was positive for CD29, CD44, CD73, CD105, and CD90, which are important cell surface markers of MSCs, but negative for CD45 and CD34, which are two specific cell surface markers of hematopoietic cells [11, 36, 37].

### 3.2. Stable Overexpression of Trx-1 in BMSCs

**Figure 2:**
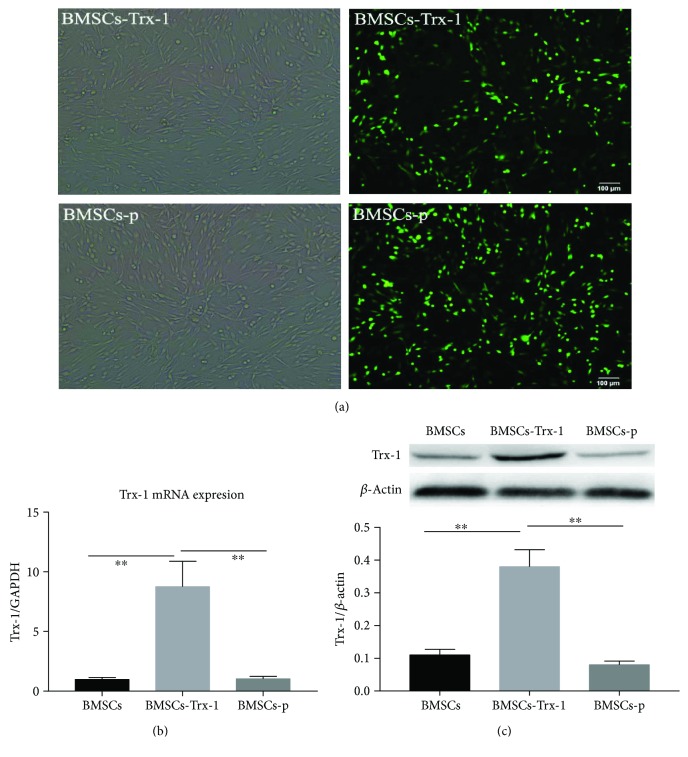
Stable overexpression of Trx-1 in BMSCs. (a) Intensive green fluorescence was observed by fluorescence microscopy (×100). (b) The mRNA levels of Trx-1 in BMSCs, BMSCs-Trx-1, and BMSCs-p. (c) Detection of Trx-1 protein expression by Western blot analysis. ^∗∗^ *P* < 0.01 compared to control. BMSCs: intact BMSCs; BMSCs-p: empty lentivirus-engineered BMSCs; BMSCs-Trx-1: Trx-1-engineered BMSCs.

For stable overexpression of Trx-1 in BMSCs, the cells were transfected with a plasmid encoding Trx-1. After the transfection and drug selection, the expression of GFP-tagged Trx-1 was confirmed by fluorescence microscopy (Figure 2(a)). Compared to control cells, we observed that BMSCs-Trx-1 exhibited an 8-fold increased Trx-1 mRNA expression and showed a 4-fold increased protein content (Figures 2(b) and 2(c)). In order to examine whether BMSCs exhibit phenotypic changes after Trx-1 transfection, the expression patterns of cell surface markers were compared between intact BMSCs and BMSCs-Trx-1. We found that there were no marked differences in the expression patterns of cell surface markers between the two cells, indicating that regardless of transfection, these cells were genetically stable (Supplement Figure 1).

### 3.3. Effects of Hyperoxia and Trx-1 Overexpression on Cell Proliferation

**Figure 3:**
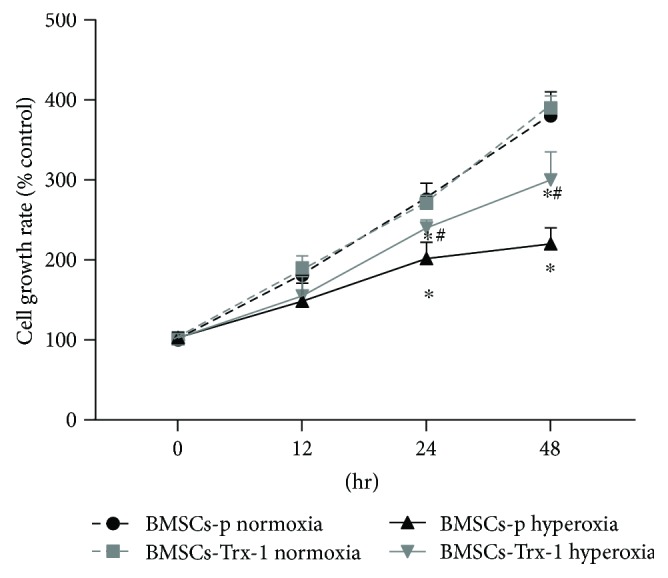
Overexpression of Trx-1 promoted proliferation of BMSCs under hyperoxic conditions. Cells with or without Trx-1 overexpression were exposed to hyperoxia for the indicated time. Cell proliferation was estimated using a CCK-8 kit. Hyperoxia treatment inhibited BMSC proliferation. However, overexpression of Trx-1 increased cell growth rate under hyperoxic conditions compared to BMSCs-p. Growth curve was generated by reading the absorbance value at 450 nm. The value was computed as percent of 0 hour. The results were expressed as mean ± SD of the results of three independent experiments, each with triplicates. ^∗^ *P* < 0.05 or 0.01 compared to normoxia control, ^#^ *P* < 0.05 or 0.01 compared to BMSCs-p under hyperoxia conditions. BMSCs-p: empty lentivirus-engineered BMSCs; BMSCs-Trx-1: Trx-1-engineered BMSCs.

The effects of hyperoxia and Trx-1 overexpression on the proliferation of BMSCs were assessed by CCK-8 assay kit. As shown in Figure 3, hyperoxia treatment inhibited BMSC proliferation in a time-dependent manner. Compared to cells cultured in normoxia, the growth rate of the hyperoxia-treated cells was significantly inhibited starting at 24 hours. After 48 hours of hyperoxia exposure, BMSCs-p proliferation was inhibited by more than 40%. However, BMSCs-Trx-1 proliferation was only inhibited by 23% at 48 hours, suggesting that Trx-1 overexpression significantly increased the proliferation of cells under hyperoxic conditions.

### 3.4. Assessment of Cell Apoptosis

**Figure 4:**
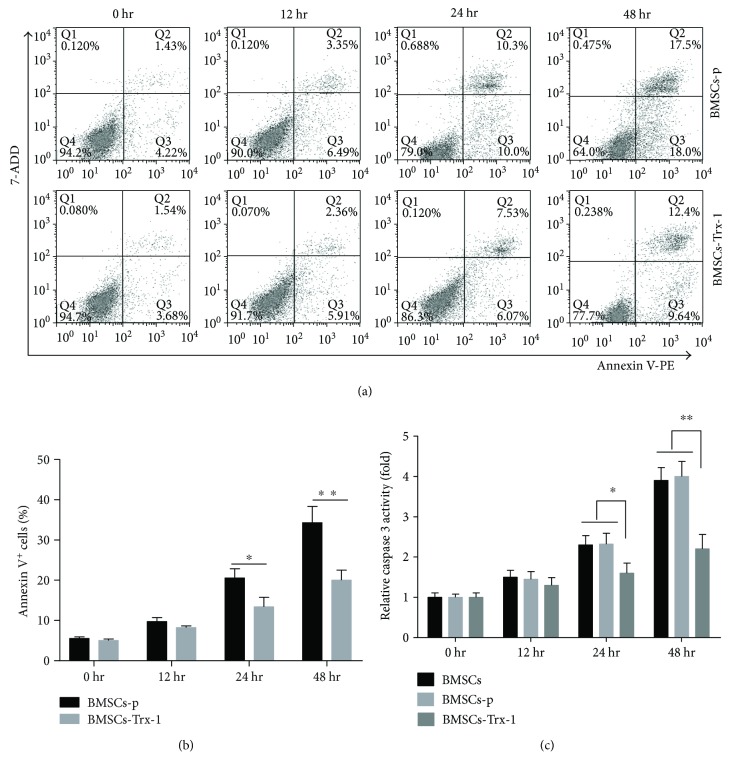
Effect of Trx-1 on cell apoptosis in BMSCs. Cells were exposed to hyperoxia for 0, 12, 24, and 48 hours and were stained with annexin V-PE/7-ADD before flow cytometry analysis. (a) Dot plots of flow cytometry analysis. Intensity of 7-ADD staining (*y*-axis) was plotted versus annexin V intensity (*x*-axis). Numbers indicate percent in each region. (b) The graph shows the percentage of apoptosis as defined by annexin V^+^. The results are representative of 3 independent experiments. (c) Caspase 3 activity. Caspase 3 activity was measured by the caspase 3 activity kit. Bar graphs represent the relative expression of caspase 3 activity calculated from each group. The results are representative of 3 independent experiments. ^∗^ *P* < 0.05, ^∗∗^ *P* < 0.01 compared with the BMSCs-p group or BMSCs. BMSCs: intact BMSCs; BMSCs-p: empty lentivirus-engineered BMSCs; BMSCs-Trx-1: Trx-1-engineered BMSCs.

To investigate the effects of hyperoxia and Trx-1 overexpression on the induction of apoptosis in BMSCs, we labeled cells with annexin V-PE, a marker of early apoptosis, and with 7-ADD, a marker of necrosis, to be analyzed via flow cytometry. As shown in Figures 4(a) and 4(b), hyperoxia induced apoptosis in a time-dependent manner in cells regardless of Trx-1 overexpression. The percent of apoptotic cells, as seen by annexin V^+^ staining, was increased in hyperoxia-treated BMSCs-p (about 20% at 24 hours and 35% at 48 hours). Trx-1 overexpression inhibited this hyperoxia-induced cell apoptosis, as seen by the decreased percent of annexin V^+^ cells (about 13% at 24 hours and 20% at 48 hours).

### 3.5. Trx-1 Inhibits Caspase 3 Activity

Caspase 3 is one of the key mediators of apoptosis; so, to further evaluate the antiapoptotic effects of Trx-1, we monitored caspase 3 activity using the Caspase 3 Activity Assay Kit. Results showed that caspase 3 activity increased when cells were treated with hyperoxia (Figure 4(c)). Compared to BMSCs and BMSCs-p, overexpression of Trx-1 in BMSCs-Trx-1 reduced caspase 3 activities under hyperoxic conditions, with the largest difference seen at 48 hours (about 50% was inhibited compared to BMSCs-p).

### 3.6. Trx-1 Reduced the Intracellular Total ROS and Hydrogen Peroxide Formation under Hyperoxic Conditions

**Figure 5:**
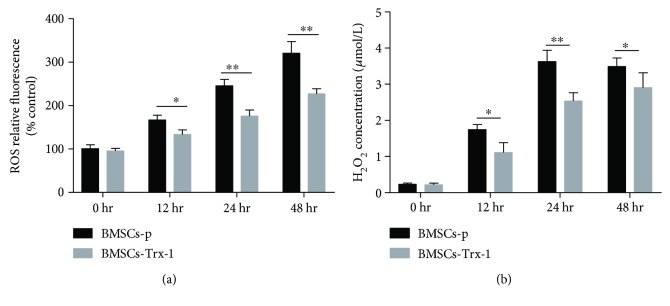
Effects of Trx-1 on intracellular ROS levels in BMSCs. (a) Intracellular ROS production was measured with CellROX deep red reagent, which can detect total ROS and was not the target particular species. The relative fluorescence intensity was expressed as % compared to control cells (BMSCs-p at 0 hr). (b) The level of intracellular H_2_O_2_ was measured using hydrogen peroxide assay kit. Experiments were repeated three times. ^∗^ *P* < 0.05, ^∗∗^ *P* < 0.01 versus the corresponding group. BMSCs-p: empty lentivirus-engineered BMSCs; BMSCs-Trx-1: Trx-1-engineered BMSCs.

To further explore the mechanisms by which Trx-1 reduces hyperoxia-induced BMSC injury, intracellular ROS levels were measured by flow cytometry analysis of cells stained with CellROX deep red reagent. As shown in Figure 5(a), exposure of BMSCs to hyperoxia markedly increased the generation of ROS in a time-dependent manner (increased 2-fold at 48 hours). Compared with the BMSCs-p group, Trx-1 overexpression markedly decreased the hyperoxia-induced ROS formation in the BMSCs-Trx-1 group (decreased 20%~30% versus BMSCs-p control).

Subsequently, the level of intracellular H_2_O_2_ was determined, as it is an important ROS. The results showed that H_2_O_2_ production was increased with longer hyperoxia exposure (normoxia: 0.23 *μ*M and hyperoxia: 3.5 *μ*M at 48 hours) (Figure 5(b)). Trx-1 overexpression inhibited hyperoxia-induced H_2_O_2_ generation in BMSCs. The strongest inhibition happened at 12 hours (the percent inhibition exceeded 35%).

### 3.7. Effects of Trx-1 on Antioxidant Enzyme Activities in BMSCs

**Figure 6:**
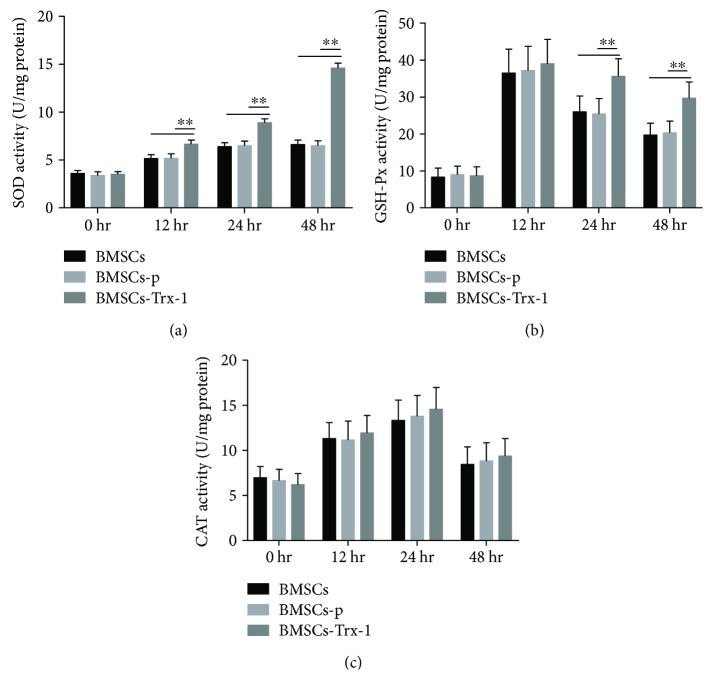
Effects of Trx-1 overexpression on antioxidant enzyme activities in BMSCs under hyperoxic conditions. (a) Superoxide dismutase (SOD) activities were measured using the SOD assay kit. (b) Glutathione peroxidase (GSH-Px) activities were measured using the glutathione peroxidase assay kit. (c) Catalase (CAT) activities were measured using the CAT assay kit. Data are representative of duplicate samples from five experiments. ^∗∗^ *P* < 0.01. BMSCs: intact BMSCs; BMSCs-p: empty lentivirus-engineered BMSCs; BMSCs-Trx-1: Trx-1-engineered BMSCs.

The activities of three major endogenous antioxidant enzymes (SOD, CAT, and GSH-Px) were then analyzed in the three BMSC lines (BMSC, BMSCs-p, and BMSCs-Trx-1). After treatment with hyperoxia, significant increases in SOD and GSH-Px activities were detected in these three groups of BMSCs. As shown in Figure 6(a), Trx-1 overexpression in BMSCs further increased SOD activity compared to BMSCs with normal Trx-1 expression. In the three BMSCs, GSH-Px activity was increased by a similar degree after hyperoxia exposure for 12 hours (Figure 6(b)). After 24 hours of hyperoxia exposure, the activities of GSH-Px began to decrease gradually. However, when compared to BMSCs and BMSCs-p, BMSCs-Trx-1 with Trx-1 overexpression upregulated GSH-Px activity after 24 hours and 48 hours of hyperoxia exposure. Trx-1 was not found to have any effect on CAT activity (Figure 6(c)).

### 3.8. Trx-1 Has No Effect on Cytokine Secretion from BMSCs

Recently, an increasing number of studies has shown that the protective effects of BMSC transplantation may be predominantly mediated by paracrine, rather than regenerative, mechanisms [7]. To determine whether Trx-1 exerts its cytoprotective effects by regulating cytokine secretion from BMSCs, the levels of EGF, KGF, and HGF in the cell culture medium were assayed by ELISA. Our results showed that Trx-1 overexpression only slightly increased the levels of secreted EGF, KGF, and HGF. However, these differences were not statistically significant across the three groups (Supplement Figure 2).

### 3.9. Effects of Trx-1 on the ASK1/P38 MAPK Pathway

**Figure 7:**
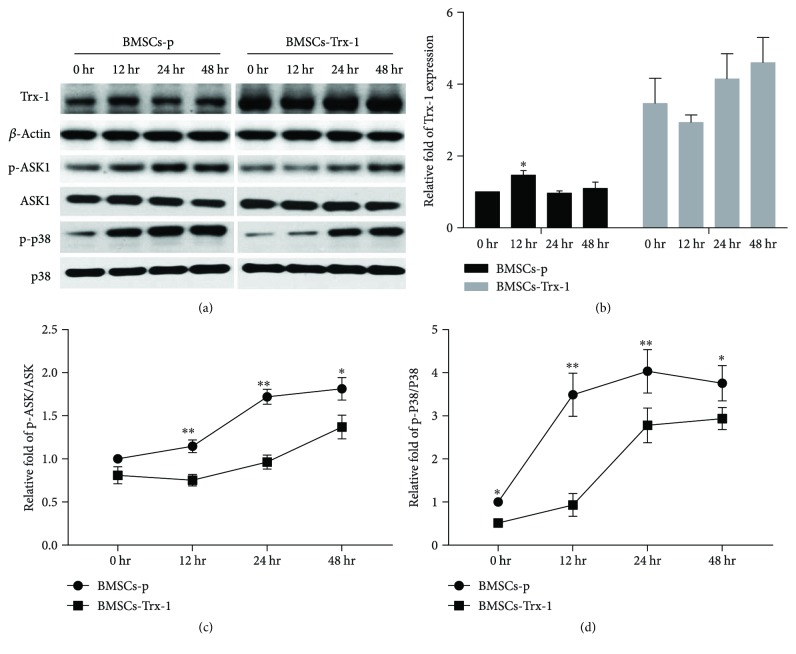
Western blot results. Trx-1, phospho-ASK1, total ASK1, phospho-p38, and total p38 expressions were detected by Western blotting. (a) Representative Western blot bands. (b) Trx-1 densitometric analysis. (c) p-ASK/ASK densitometric analysis. (d) p-38/P38 densitometric analysis. Data are representative of three independent experiments. BMSCs-p: empty lentivirus-engineered BMSCs; BMSCs-Trx-1: Trx-1-engineered BMSCs. ^∗^ *P* < 0.05; ^∗∗^ *P* < 0.01 versus the corresponding group.

To investigate the influence of hyperoxia on Trx-1 expression, we compared the protein levels of Trx-1 after different hyperoxia exposures. As shown in Figures 7(a) and 7(b), after 12-hour hyperoxia exposure, Trx-1 expression was significantly increased (about 50%). However, Trx-1 expression returned to almost normal levels after 24 hours of hyperoxia treatment in BMSCs-p cells. Trx-1 overexpression did not change throughout 0 to 48 hours of hyperoxia treatment in BMSCs-Trx-1 cells.

Hyperoxia-induced activation of ASK1 was confirmed by a significant increase in phospho-ASK1 levels as detected by Western blotting, which was significantly inhibited by Trx-1 overexpression (Figures 7(a) and 7(c)). We next examined whether p38, a potential downstream signal of ASK1, was involved in hyperoxic cell injury pathogenesis. As shown in Figures 7(a) and 7(d), activation of p38 via phosphorylation was upregulated under hyperoxic conditions as measured by phoshpo-p38, with levels peaking at 24 hours (about 4-fold upregulation compared to 0 hour). Trx-1 overexpression in BMSCs significantly suppressed the phosphorylation of p38.

## 4. Discussion

Bone marrow-derived mesenchymal stem cells (BMSCs) are easily isolated and amplified, are immunologically tolerant, and have multilineage potential, which makes them an ideal candidate for intense investigation as a cell-based therapeutic strategy for many kinds of diseases, including BPD [38]. The isolated primary BMSCs in this study have properties of mesenchymal stromal cells, according to the criteria of the International Society for Cellular Therapy (ISCT) [39], such as being spindle shaped, plastic adherent, CD29+, CD44+, CD73+, CD105+, CD90+, CD45−, and CD34− and having multipotent differentiation (Figure 1).

In rodent models of BPD, MSC administration by intravenous injection or intratracheal instillation resulted in stimulation of lung tissue repair, decreased vascular remodeling, pulmonary hypertension, and right ventricular hypertrophy [40]. Furthermore, in experimental models of BPD, intratracheal administration of MSC-conditioned medium resulted in similar short-term regenerative effects as those with administration of MSCs [41]. MSCs play protective effects in BPD, not only by engraftment and differentiation into specific lung cell types but also by secreting several anti-inflammatory cytokines and growth factors that affect cell proliferation, differentiation, and survival [7].

Despite visible advances in the field of MSC-based therapy, the reported functional improvements are generally modest partly because of the low cellular survival rate [7, 11]. Studies have indicated that pathophysiological environmental conditions, including oxidative stress and inflammation, can lead to poor viability and apoptosis of MSCs [42]. MSCs are characterized by the requirement of a low-oxygen tension environment, about 2%–8% O_2_ [43]. In view of these observations, together with the fact that oxygen toxicity plays a critical role in the lung injury process leading to BPD [44], it is suggested that hyperoxia may be the first factor to threaten MSC survival in BPD. In the present study, we demonstrated that hyperoxia inhibited BMSC proliferation by 26.8% at 24 hours and 42% at 48 hours (Figure 3). Consistent with this result, hyperoxia treatment induced BMSC apoptosis in 20% cells at 24 hours and 35% cells at 48 hours (Figure 4). These results suggested that hyperoxia-induced injury plays a key role in BMSC death. Therefore, strategies to improve BMSC tolerance to hyperoxic conditions might improve the survival of transplanted cells and consequently increase their beneficial therapeutic effects on hyperoxia-induced injury.

Recently, diverse approaches involving the genetic modification of MSCs have been undertaken to increase survivability [45]. The thioredoxin system has been demonstrated to play a key role in modulating redox signaling pathways and can be induced by a wide variety of stress conditions, such as oxidative stress, ultraviolet irradiation, *γ*-rays, hypoxia, lipopolysaccharide, and viral infections [46–48]. In the present study, we demonstrated that hyperoxia also could induce Trx-1 expression in BMSCs, but only within a short time frame (12 hours) (Figures 7(a) and 7(b)). Our previous studies have shown that exogenous addition of Trx can prevent hyperoxia-induced alveolar type II epithelial cell apoptosis [20]. Furthermore, cell injury in A549 cells, a lung epithelial adenocarcinoma cell line, has been shown to be significantly aggravated by Trx-specific siRNA under hyperoxic conditions [49]. In other cells, Trx-1 redox signaling was reported to regulate H1299 cell survival in response to hyperoxia [50]. Hyperoxic impairment of Trx-1 has a negative impact on peroxiredoxin-1 and HSP90 oxidative responses. These studies have led to the idea that Trx-1 can promote MSC survival in various conditions. Suresh et al. have experimented with overexpression of Trx-1 to increase engrafted MSC survivability in the treatment of cardiac failure [8]. Their results showed that following myocardial infarction, treatment with MSCs transfected with Trx-1 overexpression vectors increased their capacity for survival, proliferation, and differentiation, which promoted heart function and decreased fibrosis when compared to that with untransfected MSCs. Based on a similar premise, our present study aims to determine if Trx-1 overexpression can attenuate hyperoxia-induced BMSC injury using BMSCs we successfully engineered to overexpress the Trx-1 gene. Additionally, we confirmed that Trx-1 overexpression did not change BMSCs' genetic stability (Supplement Figure 1).

To examine the effect of Trx-1 on BMSC survival under hyperoxic conditions, cell proliferation rates and apoptosis were estimated in rat BMSCs with or without Trx-1 overexpression. As shown in Figures 3 and 4, BMSCs-Trx-1 showed increased cell proliferation rates and decreased apoptosis under hyperoxic conditions compared to BMSCs-p control, suggesting that Trx-1 overexpression causes cells to be more resistant to hyperoxic stress. Caspases, a family of cysteine proteases, are expressed in almost all cell types as inactive proenzymes. Caspase activation is thought to be a key step in the genesis of apoptosis. Caspases are either initiators or executioners, and caspase 3 is known to play a key role in the execution of apoptosis [51]. To test whether caspase 3 was involved in hyperoxia-induced apoptosis, we probed for caspase 3 activity. The results showed that caspase 3 activity was increased more than 2-fold after 24-hour hyperoxia treatment and almost 4-fold after 48-hour hyperoxia treatment compared to those of the control-untreated cells (0 h). These results indicated that hyperoxia-induced BMSC apoptosis is, at least in part, caspase 3 dependent. We found that this hyperoxia-induced activation of caspase 3 was strongly inhibited by Trx-1 overexpression. These results, together with the annexin V stain assay showing a decrease in apoptosis, suggest that Trx-1 inhibited hyperoxia-induced BMSC apoptosis mainly through a caspase 3-dependent pathway.

The effects of hyperoxia on cellular function and survival have been widely held to be secondary to the generation of ROS. It has been demonstrated that ROS act as upstream signaling molecules that initiate cell death under hyperoxic conditions [52]. In our study, hyperoxia exposure resulted in an increase of intracellular ROS. However, Trx-1 overexpression could partly reverse such effects of hyperoxia (Figure 5).

H_2_O_2_ is a crucial ROS that is involved in cell signaling but can alter the intracellular redox environment when produced in excess amounts, leading to many pathophysiological conditions [53]. During exposure to hyperoxia, production of ROS is seen through the increased release of H_2_O_2_ by lung mitochondria and microsomes [54]. Accumulating evidence suggests that hyperoxia promotes intracellular H_2_O_2_ accumulation, with H_2_O_2_ playing a key role in the oxidative stress-induced injury from ROS [55]. It has been confirmed that the Trx system, which is composed of a NADPH-dependent thioredoxin reductase (TrxR) and Trx, provides electrons to thiol-dependent peroxidases (peroxiredoxin (Prx)) to directly remove H_2_O_2_ [53]. In the present study, we observed increased H_2_O_2_ generation in hyperoxia-exposed BMSCs, while Trx-1 overexpression decreased H_2_O_2_ generation under hyperoxic conditions. Additionally, compared to the total ROS generated, H_2_O_2_ was more strongly induced by hyperoxia, which suggests that H_2_O_2_ is the main source of intracellular ROS under hyperoxic conditions. However, more evidence is needed to confirm this hypothesis.

**Figure 8:**
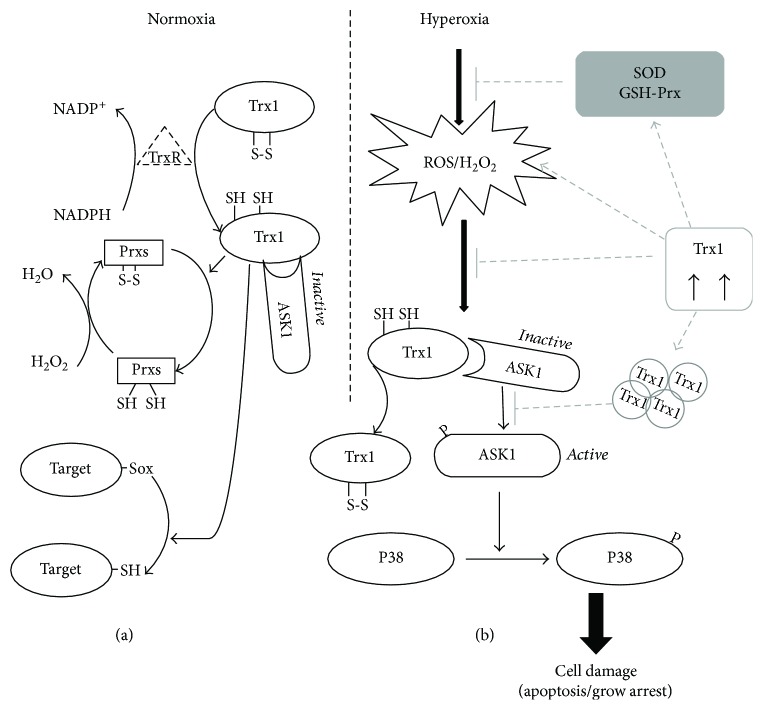
A schematic model of the regulation of the ASK1/P38 signal pathway by Trx-1. (a) The Trx-1 system contains NADPH, TrxR-1, and Trx-1. The oxidized Trx-1 (inactive form) is transformed to the active and reduced form of Trx-1 by receiving electrons from NADPH coenzyme in the presence of TrxR-1. Prxs reduce H_2_O_2_ to H_2_O using electrons from the active Trx-1. The active Trx-1 also regulates redox signals by reducing many other target proteins with disulfide bonds. ASK1 constantly forms an inactive complex with reduced Trx-1 under normoxic conditions. (b) Exposure of BMSCs to hyperoxia leads to elevated ROS and H_2_O_2_ production, which leads to oxidative stress. However, oxidized Trx-1 is dissociated from ASK1 in response to oxidative stress and subsequent activation of ASK1. Activated ASK1 in turn activates the p38 pathway and induces various cellular responses, including cell apoptosis and differentiation inhibition. Trx-1 overexpression promoted BMSC survival under hyperoxic conditions through elevation of antioxidant activities, reduction of ROS and H_2_O_2_ generation, and subsequent inhibition of the ASK1/P38 signaling pathway.

As mentioned earlier, ROS are not only cytotoxic products from the external and internal environment but are also important mediators of redox signaling. Therefore, Trx acts as an antioxidant to maintain the balance of the thiol-related redox status and thus plays a pivotal role in the regulation of redox signaling and cell survival and death [48]. Trx-1 is known to regulate several transcription factors such as NF-*κ*B, p53, and Ref-1, as well as some apoptotic factors like ASK1 [56–58]. ASK1 is a member of the mitogen-activated protein kinase kinase kinase (MAPKKK) group, which can be activated by various stresses such as oxidative stress, which can then activate caspase 3 and promote apoptosis [59]. As such, ASK-1 is necessary for ROS-induced cell death and inflammation [60]. Fukumoto et al. reported that deletion of ASK1 protects against hyperoxia-induced acute lung injury [61]. As shown in Figure 7, we confirmed that the activity of ASK1 was upregulated by hyperoxia in a time-dependent manner. It has been shown that Trx is a negative regulator of ASK1 [56].  Figure 8 shows that in resting cells, ASK1 forms an inactive complex with reduced Trx-1, but oxidation of Trx-1 leads to the dissociation of Trx-1 from ASK1, switching the ASK1 to an active kinase [48]. It has also been reported that overexpression of the Trx in endothelial cells induces ASK1 ubiquitination and degradation [16]. To demonstrate whether overexpression of Trx-1 protects BMSCs from hyperoxia-induced injury via inhibition of the ASK-1 signaling pathway, we determined the activation status of ASK1 and its downstream proapoptotic factor, the p38 MAP kinase. We did not observe obvious changes of the total ASK1 level but results demonstrated that Trx-1 overexpression inhibited hyperoxia-induced ASK1 activation. The activation of p38 has also been shown to be associated with hyperoxia-induced cell damage [62]. Previously, we demonstrated that Trx can protect alveolar epithelial cells from hyperoxia-induced damage via decreasing p38 activation [20]. It was reported that ASK1 is required for the sustained activation of JNK/p38 MAP kinases, leading to apoptosis [63]. In the present study, we showed that Trx-1 overexpression in BMSCs inhibited hyperoxia-induced p38 activation. Taken together, these results indicate that inhibition of the ASK1/P38 pathway was involved in the mechanism of Trx-1-mediated protection of BMSCs from hyperoxia-induced injury (Figure 8). Recently, several studies suggest that the protective effects of stem cell transplantation might be predominantly mediated by a paracrine mechanism [7, 38] and that growth factors such as VEGF, HGF, and KGF are critical in mediating the protective effects of MSCs against hyperoxic lung injury [64]. With regard to these growth factors, we found no difference between our three BMSC cell lines under hyperoxic conditions in vitro (Supplement Figure 2). Based on this, Trx-1 appears to protect BMSCs from hyperoxia-induced injury independently of paracrine growth factors. However, whether Trx-1 overexpression affects the therapeutical effect of BMSC via paracrine growth factors in vivo will need further studies.

Other mechanisms, such as changes in expression of antioxidant enzymes, may also be involved in the cellular response of Trx-1-overexpressing BMSCs against hyperoxia. Several studies reported greater MSC survival from oxidative stress injury via increased activities of antioxidant enzymes [65, 66]. Three of the primary antioxidant enzymes in oxygen-metabolizing mammalian cells believed to be necessary for cell survival are SOD, CAT, and GSH-Px. SOD is a metalloenzyme that catalyzes the dismutation of superoxide anion into O_2_ and H_2_O_2_. Subsequently, H_2_O_2_ is reduced to H_2_O by GSH-Px in the cytosol or by CAT in peroxisomes or cytosol [67]. In our study, we demonstrated that Trx-1 overexpression enhanced the activities of the antioxidant enzymes SOD and GSH-Px, resulting in maintenance of relatively low intracellular levels of ROS and H_2_O_2_, as shown in Figures 5 and 6. CAT is a common enzyme found in nearly all living organisms exposed to oxygen. It is a very important enzyme in the biological defense system. Zhang et al. demonstrated CAT transduction was able to increase MSC viability and promote ischemia-induced angiogenesis [68]. However, we did not observe CAT activity to be affected in this study. The mechanism involved by which Trx-1 selectively affects different antioxidant enzymes requires further studies.

In conclusion, our results indicate that hyperoxia exposure induced BMSC apoptosis, which may contribute to the low survival rate of transplanted BMSCs and that Trx-1 overexpression has a significant effect on improving the survival rate of BMSCs. The summary of our results in the study is shown in Figure 8.

## Supplementary Materials

Supplementary 1 Supplement Figure 1: BMSCs-Trx-1 remains MSC characteristics. In order to examine the possibility of MSC phenotypic changes after Trx-1 transfection, the expression patterns of cell surface markers were compared between intact BMSCs and BMSCs-Trx-1.

Supplementary 2 Supplement Figure 2: Trx-1 did not affect cytokine secretion from BMSCs. The levels of secreted EGF (A), KGF (B), and HGF (C) were measured by Elisa kits. Data are representative of duplicate samples from three experiments.
